# Amplifying the efficacy of ALA-based prodrugs for photodynamic therapy using nanotechnology

**DOI:** 10.3389/fphar.2023.1137707

**Published:** 2023-02-27

**Authors:** Liang Lou, Shizhe Zhou, Sijia Tan, Menghua Xiang, Wei Wang, Chuang Yuan, Liqian Gao, Qicai Xiao

**Affiliations:** ^1^ School of Pharmaceutical Sciences (Shenzhen), Sun Yat-sen University and Shenzhen Campus of Sun Yat-sen University, Shenzhen, China; ^2^ Xiangya School of Pharmaceutical Sciences, Central South University, Changsha, China; ^3^ Department of Hematology, Xiangya Hospital, Central South University, Changsha, China

**Keywords:** 5-aminolevulinic acid, biotransformation, photodynamic therapy, prodrug, nanotechnology

## Abstract

5-aminolevulinic acid (ALA) is a clinically approved prodrug involved in intracellular Heme biosynthesis to produce the natural photosensitizer (PS) Protoporphyrin IX (PpIX). ALA based photodynamic therapy (PDT) has been used to treat various malignant and non-malignant diseases. However, natural ALA has disadvantages such as weak lipophilicity, low stability and poor bioavailability, greatly reducing its clinical performance. The emerging nanotechnology is expected to address these limitations and thus improve the therapeutic outcomes. Herein, we summarized important recent advances in the design of ALA-based prodrugs using nanotechnology to improve the efficacy of PDT. The potential limitations and future perspectives of ALA-based nanomedicines are also briefly presented and discussed.

## 1 Introduction

Photodynamic therapy (PDT) is a clinically approved modality involving the combination of a photosensitizer (PS) and molecule oxygen to produce cytotoxic reactive oxygen species (ROSs), primarily singlet oxygen (^1^O_2_), thereby inducing cell death (Xiao et al., 2018; Li Q et al., 2020). The unique mechanism of action makes PDT safe, efficient, reproducible and minimally invasive. The inherent fluorescent features of PSs endow them with capabilities in fluorescence-guided surgery and diseases diagnosis (Ji et al., 2021; Xiang et al., 2021). Besides, with rich experience in anti-tumor PDT, a large number of researchers have applied PDT to fight against microorganisms, especially drug-resistant bacteria (Liu et al., 2016; Jia et al., 2019; Xiao et al., 2020; Xiao et al., 2021a).

**FIGURE 1:**
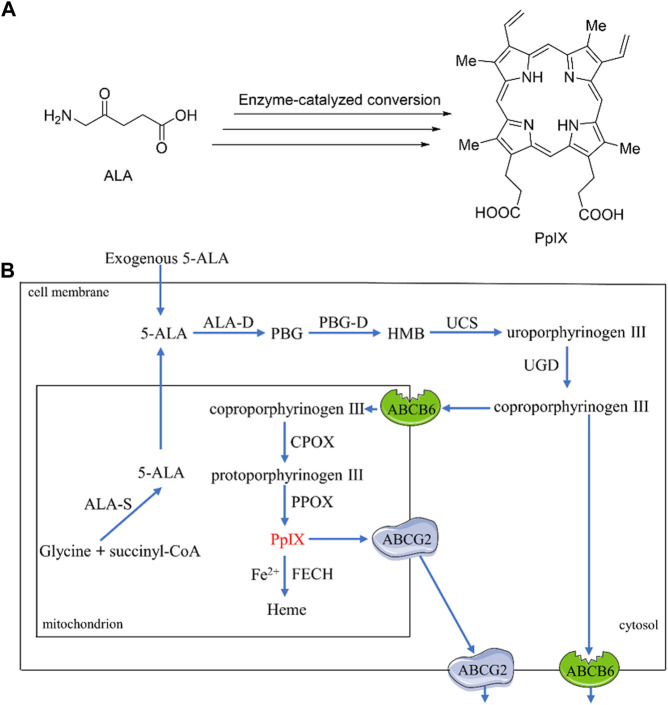
**(A)** Structures of ALA and PpIX; **(B)** A simplified mechanism of enzyme-catalyzed bio-transformation from ALA to PpIX.

5-aminolevulinic acid (ALA) is a clinically approved prodrug of natural PS protoporphyrin IX (PpIX) (Figure 1A). Upon systematical or topical administration, ALA molecules penetrate into cells, where they participate in the biosynthesis of Heme and produce PpIX (Figure 1B). Briefly, the conversion involves a total of eight steps catalyzed by eight enzymes, of which four steps occur in the mitochondria and another four steps occur in the cytosol (Ponka, 1999). Firstly, ALA presented in the cytosol, including exogenous and endogenous molecules, are condensed to generate porphobilinogen (PBG) under catalysis by ALA dehydratase (ALA-D). Next, four units of PBG *via* mediation by porphobilogen deaminase (PBG-D) are connected to form tetrapyrrole intermediate hydroxymethylbilane (HMB), which is then cyclized to provide uroporphyrinogen III upon activation by uroporphyrinogen III cosynthase (UCS). The resulting uroporphyrinogen III is converted to coproporphyrinogen III through decarboxylation by uroporphyrinogen decarboxylase (UGD), and then migrates to mitochondria, where it is oxidized to protoporphyrinogen III by coproporphyrinogen III oxidase (CPOX). Finally, protoporphyrinogen III is oxidized by protoporphyrinogen III oxidase (PPOX) to produce PpIX. Further conversion of PpIX to the downstream substrate of Heme requires the participation of Fe^2+^ and a rate-limiting enzyme ferrochelatase (FECH). The endogenous ALA molecules are generated in mitochondria from succinyl-CoA and glycine mediated by ALA synthase (ALA-S). Collectively, the intracellular PpIX content under normal physiological condition is tightly regulated by the negative feedback of Heme itself. Nevertheless, external administration of ALA can make cells produce abundant PpIX, which cannot be rapidly converted into Heme by FECH, thereby accumulating within cells and making ALA one of the most successful prodrugs in cancer treatment (Vallejo et al., 2021).

ALA-based PDT has been widely used to treat a variety of neoplastic and non-neoplastic diseases, including bladder cancer, basal cell carcinoma, head and neck cancer, and skin disorders such as actinic keratosis, psoriasis, acne and Bowen’s disease (Allison and Sibata, 2010; Xiao et al., 2018). ALA-assisted fluorescence diagnosis has been used for visualization of bladder, lung and skin cancers (Peng et al., 1997). The clinically available ALA and its derivatives (e.g., methyl ester derivative, MAL; hexyl ester derivative, HAL) and their associated indications are summarized in Table 1.

**TABLE 1:** Clinically used ALA and its derivatives in PDT.

Compound	Trade name	Indications	Approved countries	Ref(s)
ALA	Levulan^®^ or Ameluz^®^	Actinic keratosis, Human Papilloma Virus, head and neck cancer, etc.	United States, Canada, Europe, Japan, China, etc.	Jeffes (2002), Gupta et al. (2017)
	Gliolan^®^	Detection of malignant glioma, etc.	Europe, Canada, Japan, etc.	Krammer and Plaetzer (2008)
MAL	Metvix^®^	Actinic keratosis, Basal cell carcinoma, Bowen’s disease, etc.	United States, Canada, Europe, Australia, etc.	Gardlo and Ruzicka (2002)
HAL	Hexvic^®^ or Cysview^®^	Detection of recurrent bladder cancer, etc.	United States, Europea, New Zealand, etc.	Compérat et al. (2011), Pietzak (2017)

Increasing the drug concentration in the target tissue is beneficial to improve the therapeutic efficacy. The intracellular biosynthesis of PpIX is mediated by multiple enzymes and several important membrane transporters, including ATP-binding cassette sub-family B member 6 (ABCB6) and ATP-binding cassette sub-family G member 2 (ABCG2) (Yamamoto et al., 2020) (Figure 1B). ABCB6 present on the mitochondrial outer membrane are involved in transporting coproporphyrinogen III into mitochondria (Krishnamurthy et al., 2006), whilst those present in the cell membrane (Paterson et al., 2007) can transport coproporphyrinogen III out of the cells (Matsumoto et al., 2015). The net effect of ABCB6 on PpIX level is thus depending on the relative activity of ABCB6 in mitochondria and cell membrane. Besides, ABCG2, an efflux transporter present in mitochondria as well as cell membranes, is able to induce extracellular transport of PpIX synthesized in mitochondria (Kobuchi et al., 2012). Therefore, to improve the accumulation of PpIX in cells can generally be achieved by 1) enhancing the activities of CPOX by using upregulated molecules such as methotrexate (MTX) and vitamin D (Ortel et al., 2002; Sinha et al., 2006); 2) using Fe^2+^ chelators such as 1,2-diethyl-3-hydroxypyridin-4-one hydrochloride (CP94) or deferoxamine (DFO) to lower the conversion of PpIX to Heme (Richardson et al., 1994; Pye and Curnow, 2007); and 3) using ABCG2 inhibitors such as Fumitremorgin C (Robey et al., 2005), Ko143 (Palasuberniam et al., 2015), sunitinib (Mansi et al., 2022) or gefitinib (Palasuberniam et al., 2021) to reduce PpIX efflux.

However, the methods descried above are not cell-selective in accumulation of PpIX, and thus cannot distinguish tumor cells from normal cells. The inherent limitations of natural ALA such as weak lipophilicity, low stability and poor bioavailability, greatly reduce its clinical efficiency. The recently emerged nanotechnology provides new strategies to address these deficiencies, because nanocarriers-assisted drug delivery affords outstanding tumor selectivity, well-controlled drug release and good biocompatibility (Sun et al., 2020; Ji et al., 2022). Notably, nanomedicines are able to improve the therapeutic efficacy by combining multiple therapies into one platform (Ding et al., 2019). In this work, we summarized the important recent advances and strategies for designing ALA-based prodrugs using nanotechnology for enhanced PDT. The potential limitations and future perspectives of ALA-based nanomedicine are also briefly described and discussed.

## 2 ALA-based nanoprodrugs using nanotechnology

### 2.1 Stimuli-responsive ALA nanoprodrugs

Stimuli-responsive nanomedicines are agents that can recognize the microenvironment in living systems and release therapeutic molecules to the target tissues without affecting normal areas (Mura et al., 2013; Zhang et al., 2022). Such drug delivery systems are capable of controlling drug distribution, reducing potential side effects and enhancing the therapeutic efficacy. The responsive signals include endogenous stimuli such as low pH, increased glutathione (GSH) concentration and enhanced expression of certain enzymes, as well as exogenous stimuli such as light, temperature, ultrasound, and chemicals.

#### 2.1.1 pH responsive systems

pH values in tumor tissues are usually 0.5–1.0 units lower than those in healthy tissues due to glucose consumption and lactic acid accumulation caused by rapid cell proliferation (Volk et al., 1993; Pang et al., 2016). The difference in pH is thus applied as an ideal trigger for selectively releasing therapeutics in tumor tissues and/or cells. To date, numerous of drug delivery systems with pH-sensitivity have been investigated, including nanoparticles (Gao et al., 2010), nanogels (Li et al., 2021), nanoclusters (Lin et al., 2021), liposomes (Barattin et al., 2018), and micelles (Liao et al., 2018).

**FIGURE 2:**
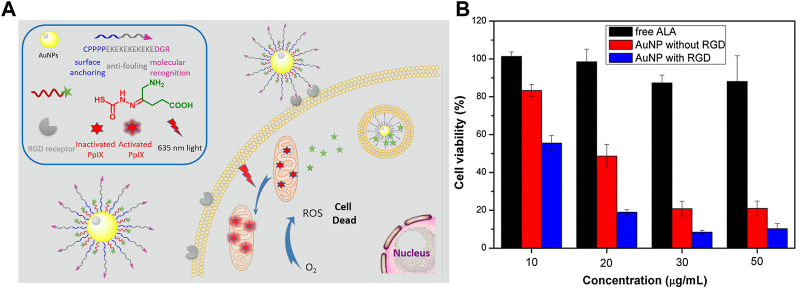
**(A)** Design and mechanism of action for peptide-modified AuNPs-loaded with pH-responsive ALA; **(B)** Cell viabilities treated with free ALA and AuNPs with or without RGD peptide under irradiation with 635 nm light. Adapted from ref. (Wu et al., 2017a). Copyright 2016 Elsevier Inc.

Gold nanoparticles (AuNPs) are a conventional type of inorganic nanoparticles used in drug delivery and biomedical imaging due to their outstanding drug loading and unique optical properties (Sperling et al., 2008; Cheng et al., 2013). The phase I clinical trials using AuNP to deliver tumor necrosis factor (TNF) have been successfully completed and no significant systematic toxicity using AuNPs was observed in animal model (Libutti et al., 2010). In 2017, Wu and coworkers reported pH-responsive zwitterionic stealth peptide-capped ALA prodrug nanoparticles for targeted PDT (Wu et al., 2017a). The nanoprodrugs were prepared by using thiolated stealth peptide sequence CPPPPEKEKEKEKEKEDGR and hydrazine-containing ALA to couple with AuNPs. The zwitterionic stealth peptide sequence EKEKEKEKEK endows nanoparticles with strong anti-fouling ability, and the RGD portion in peptide targets the RGD receptors overexpressed on the surface of cancer cells, thereby resulting in enhanced cellular uptake. The nanoparticles internalized by tumor cells release ALA in an acidic environment due to the presence of hydrazone bonds, thereby generating PpIX via a biosynthetic pathway (Figure 2A). The *in vitro* study against A549 tumor cells showed that the pH-responsive prodrug nanoparticles displayed better phototoxicity than free ALA after light irradiation (Figure 2B), suggesting that such pH-responsive ALA-loaded AuNPs have enhanced PDT effects.

Upconverting nanoparticles (UCNPs), characterized by the upconversion of lower-energy photons into high-energy photons and thereby affording deep tissue penetration and low autofluorescence, have attracted considerable attention in biomedical applications including drug delivery (Chen et al., 2014; Pickel et al., 2018). In 2014, Punjabi *et al* developed a class of biocompatible UCNPs with largely amplified red emissions by dopping high percentage of Yb into the core and coating the outer shell with biocompatible CaF_2_. These UCNPs were next conjugated with pH-responsive ALA linkages to provide ALA-conjugated UCNPs (Punjabi et al., 2014). The developed UCNPs displayed 15-fold stronger in red-emission than that of the hexagonal phased counterparts, and demonstrated significant PDT effects for the treatment of deep-set tumors (>1.2 cm). Covalent binding of ALA to UCNPs *via* hydrazone bond is able to avoid possible leakage of ALA, thus increasing ALA bioavailability and enhancing singlet oxygen generation from PpIX produced by the internalized ALA.

**FIGURE 3:**
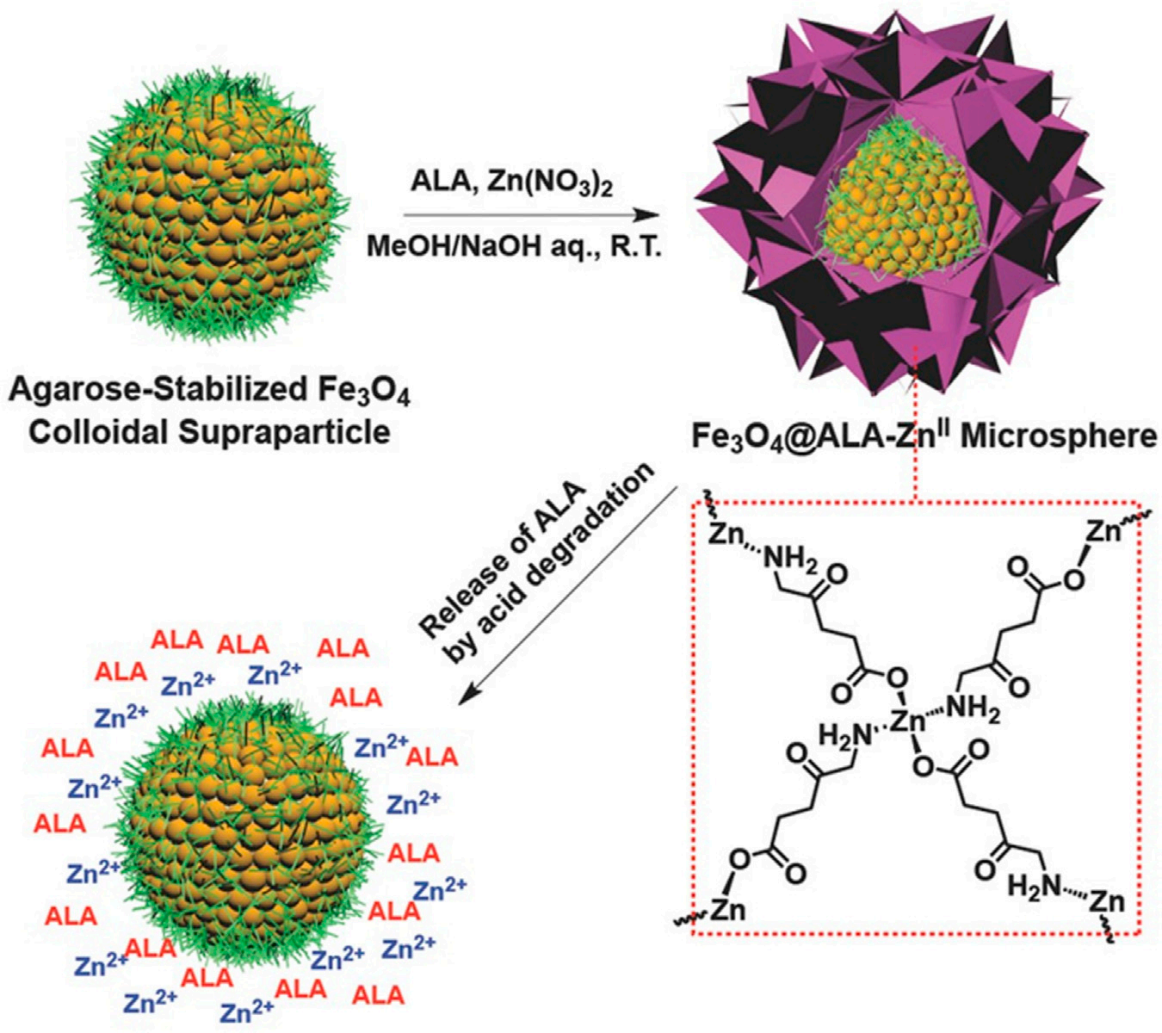
Schematic illustration of the preparation of Fe_3_O_4_ @ALA-Zn^II^ microsphere and pH-induced release of ALA. Adapted from ref. (Tan et al., 2015). Copyright 2015 WILEY-VCH Verlag GmbH & Co., KGaA, Weinheim.

In 2015, Tan and coworkers reported core/shell-structured nanoparticles (Fe_3_O_4_@ALA-Zn^II^) with agarose-stabilized magnetite colloidal supraparticles (MCSPs) as cores and 5-ALA-Zn^II^ coordination polymers as shells for PDT treatment of bladder cancer (Tan et al., 2015). The coordination polymers formed between Zn ions and the terminal NH_2_ and COOH of ALA was subsequently deposited on the surface of MNCPs to produce core/shell structure of Fe_3_O_4_@ALA-Zn^II^ (Figure 3). At normal physiological pH, ALA molecules are stabilized on the shell of MCSPs whilst being released from the dynamic coordination bonds due to the acidic microenvironment of tumors. In addition to the cell inhibition by ALA-induced PDT, Zn ions also caused additional suppression due to their ability to induce cell apoptosis.

**FIGURE 4:**
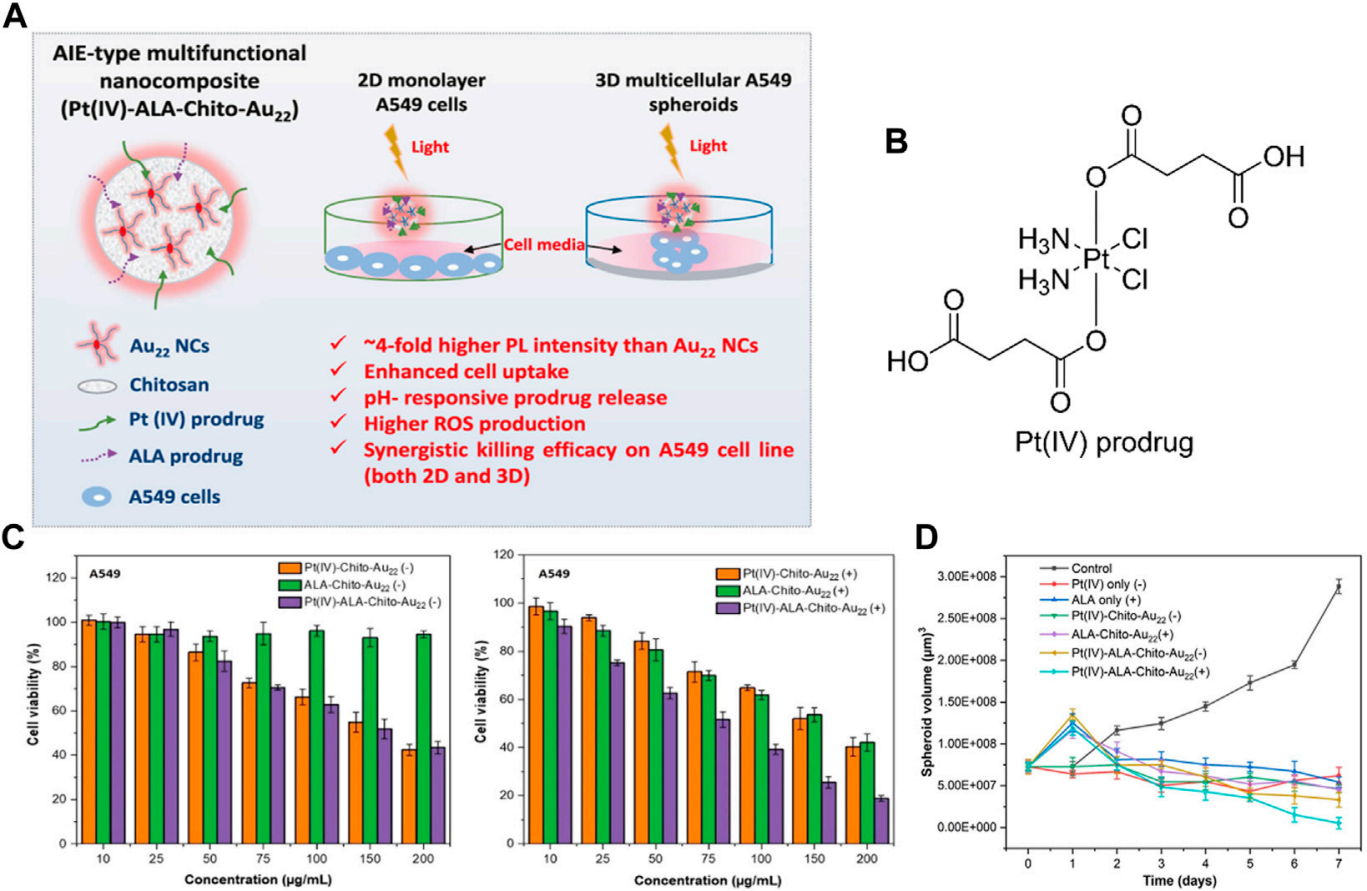
**(A)** Design of dual nanoprodrug Pt(IV)-ALA-Chito-Au_22_; **(B)** Molecular structure of the Pt(IV) prodrug; **(C)** Cell viability of A549 cells after treatment with Pt(IV)-Chito-Au_22_, ALA-Chito-Au_22_ or Pt(IV)-ALA-Chito-Au_22_ without or with light exposure; **(D)** Quantification of 3D A549 spheroid volume change in different treatment groups over 7 days. Adapted from ref. (Srinivasulu et al., 2021). Copyright 2021 American Chemical Society.

Chemo-photodynamic combined therapy is increasingly investigated in improved anticancer therapy as it can overcome tumor recurrence and multidrug resistance (Guo et al., 2018; Liu et al., 2021). In 2021, Srinivasulu et al. reported a multifunctional nanocomposite consist of Gold nanocluster (Au22), chitosan, chemotherapeutic platinum (Pt(IV)) prodrug and photodynamic ALA prodrug, denoted as Pt(IV)-ALA-Chito-Au_22_ (Srinivasulu et al., 2021). Pt(IV) and ALA prodrugs were coated on the amino-rich Au_22_-Chito nanocarriers *via* amide bonds and subsequently released in the acidic microenvironment of tumor cells (Figures 4A, B). This nanocluster-based prodrug system demonstrated good stability, enhanced cellular uptake and ROS generation. At acidic pH around 5.0, the cumulative drug release was more than 50% within 12 h. Especially, enhanced cell killing effects were observed with the dual nanoprodrug of Pt(IV)-ALA-Chito-Au_22_ than any single prodrug that conjugated with Chito-Au_22_ (Figures 4C, D). This study suggests that combining two different prodrugs into one single nanocarrier affords an alternative strategy for enhanced cancer treatment.

**FIGURE 5:**
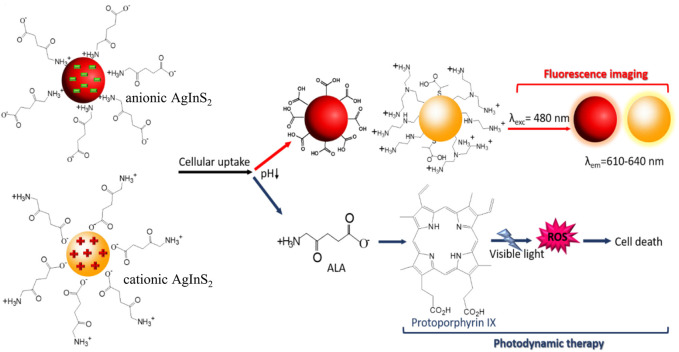
Schematic showing the design and responsive mechanism of ALA-loaded anionic and cationic AIS QDs. Adapted from ref. (Hashemkhani et al., 2022). Copyright 2022 American Chemical Society.

Quantum dots (QDs) are important semiconductor nanomaterials with fascinating optical properties and quantum confinement effects, making them potential candidates for nanoprobes and nanocarriers in biomedical applications, especially for the early detection, monitoring and treatment of certain localized diseases (Ruzycka-Ayoush et al., 2021). Recently, Hashemkhani and coworkers reported one-step aqueous synthesis of cationic and anionic silver-indium-sulfide quantum dots (AIS QDs), which were subsequently loaded with ALA through electrostatic interaction for enhanced PDT (Hashemkhani et al., 2022). The obtained AIS QDs demonstrated high quantum yields, long-term stability and excellent fluorescence imaging ability under light illumination, and thus can be used for efficient cell/tissue visualization. Importantly, these ALA-loaded AIS QDs showed sustained ALA release in the acid tumor microenvironment while protecting ALA at physiological pH, thereby achieving selective tumor destruction through intracellular bioconversion into PpIX (Figure 5). Although the delivery efficiency of cationic and anionic AIS-QDs was comparable, the anionic AIS-QDs appeared to be safer due to their relatively lower cytotoxicity.

Besides, Tong and coworkers reported dual pH-responsive ALA pseudopolyrotaxane prodrug micelles for improved PDT (Tong et al., 2016). The pH-responsive ALA fragment was prepared by conjugating ALA to α-cyclodextrin (α-CD) *via* an acid-sensitive hydrazone linkage, and the acid-sensitive PEG fragment was prepared by connecting PEG to pH-responsive CPP R6H4 (RRRRRRHHHH), thereby affording the dual pH-responsive prodrug micelles through host guest interactions between α-CD and PEG fragments. The introduction of pH-sensitive CPPs can enhance the cellular uptake of micelles in an acid tumor microenvironment. Such dual-pH responsive ALA prodrug micelles thus afford improved selectivity and new opportunities for future ALA-based photodynamic antitumor therapy.

#### 2.1.2 GSH-responsive systems

It is believed that the intracellular concentration of GSH in tumor cells is higher than that in the extracellular matrix and normal cells, and GSH-responsive prodrugs are usually designed to selectively deliver therapeutic molecules into the cytosol of tumor cells (Xiao et al., 2021b; Chen et al., 2021). Generally, GSH-mediated drug delivery can be achieved by directly introducing disulfide (S-S) bonds into the therapeutic molecules or indirectly preparing disulfide-containing carriers to encapsulate therapeutic molecules.

**FIGURE 6:**
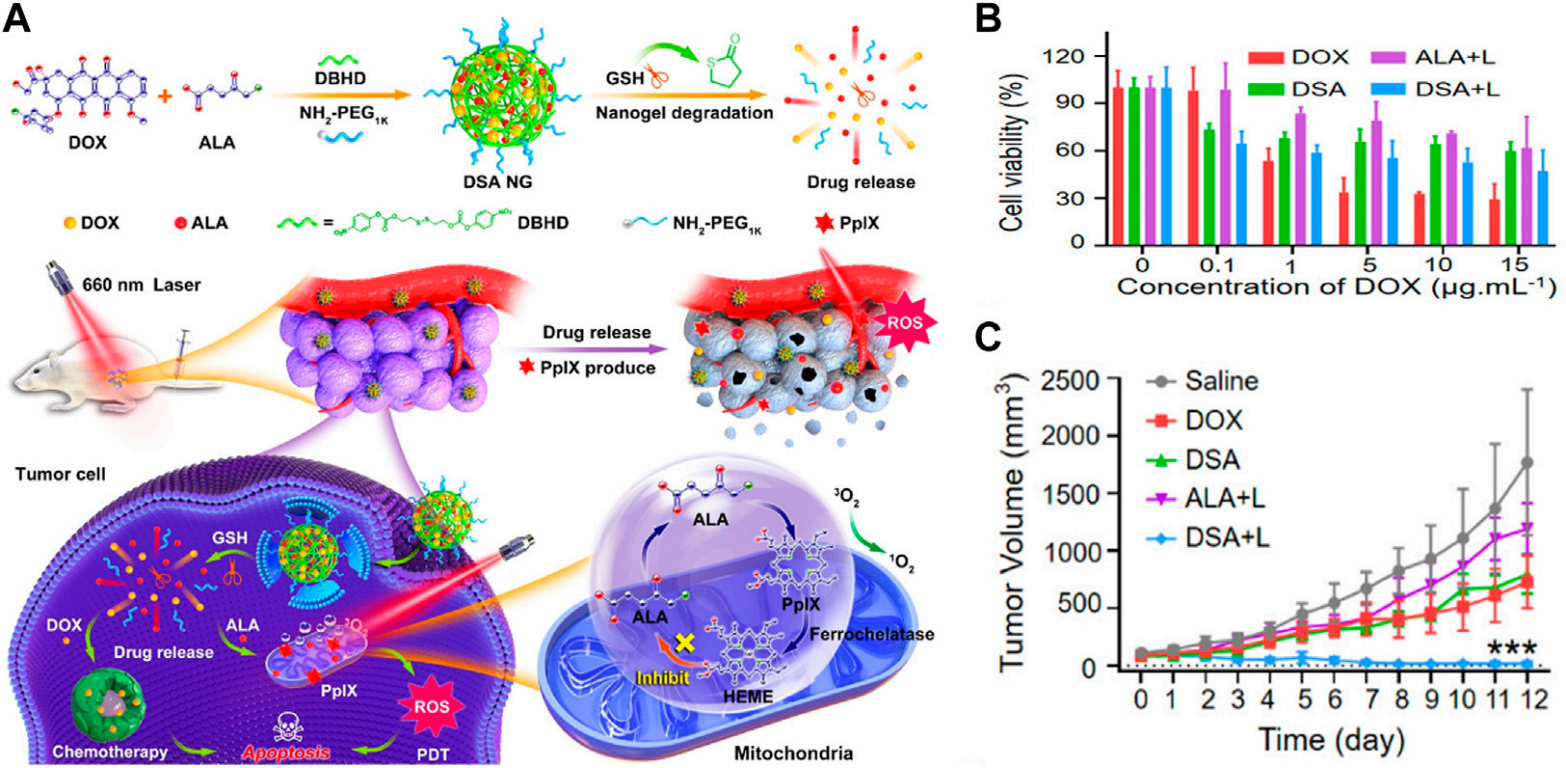
**(A)** Design and mechanism of GSH-responsive DSA NG; **(B)** Cell viability against 4T1 cells under different treatments; **(C)** Relative tumor volume over 12 days. Adapted from ref. (Wang et al., 2020b). Copyright 2020 American Chemical Society.

In 2020, Wang and coworkers reported GSH-responsive nanogels for chemo-photodynamic combined anticancer therapy (Wang et al., 2020a). Upon intermarriage with reduction-responsive cross-linker DBHD and hydrophilic chain NH_2_-PEG_1k_, ALA, and Doxorubicin (DOX) are cross-linked to form reduction-responsive nanogels (DSA NG), which has good stability, high drug loading and GSH-responsive properties (Figure 6A **)**. The internalized DSA NG release free ALA and DOX in response to high GSH concentration in tumor cells, thus inducing synergistic antitumor effects under red light irradiation (Figures 6B, C). The *in vivo* study in 4T1 tumor-bearing mice showed that DSA NG almost completely eradicated tumors (Figure 6C), suggesting GSH-sensitive nanogel might be a promising drug delivery platform to improve tumor therapy. Furthermore, this GSH-responsive prodrug strategy was also applied in chemo-immunotherapy, showing a robust antitumor immune response upon GSH activation (Ma et al., 2022).

**FIGURE 7:**
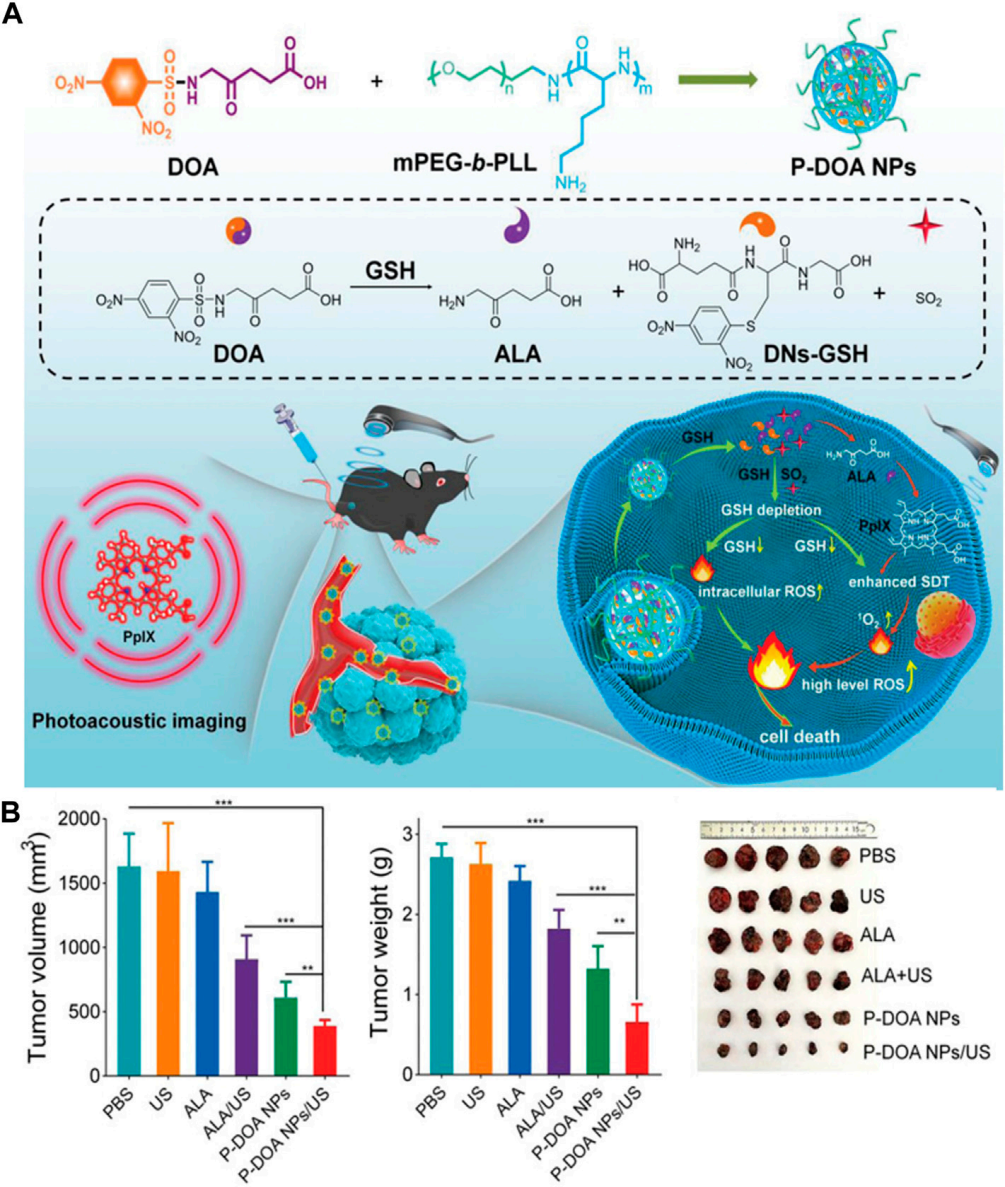
**(A)** Design and mechanism of GSH-responsive P-DOA NPs; **(B)** Relative tumor volume, weight and photographs of resected tumors in B16F10 tumor-bearing mice after treatments on day 15. (Unite States: ultrasound). Adapted from ref. (Sun et al., 2022). Copyright 2022 WILEY-VCH Verlag GmbH & Co., KGaA, Weinheim.

Sonodynamic therapy (SDT) is another relatively new cancer treatment that works by a similar mechanism of action as PDT (Gong and Dai, 2021; Zhang et al., 2021). However, high concentration of GSH in tumor cells would alleviate SDT-induced ROS damage, thus reducing the SDT effects (Kumar et al., 2022). To this end, Sun and coworkers reported a two-in-one GSH-responsive nanoprodrug for photoacoustic imaging-guided SDT for skin cancer (Sun et al., 2022). The two-in-one prodrug nanoparticles (P-DOA NPs) were co-assembled from methoxyl poly(ethylene glycol)-b-poly(l-lysine) (mPEG-b-PLL) and the GSH responsive DOA fragment was synthesized by 2,4-dinitrobenzenesulfonyl chloride (DNs-Cl) and ALA. The prodrug nanoparticles can simultaneously liberate ALA and SO_2_ under high concentration of GSH in tumor cells. The released ALA molecules were intracellularly converted into PpIX for photoacoustic imaging and SDT, whereas the released SO_2_ weakened the intracellular reductive microenvironment, thereby increasing intracellular ROS levels and resulting in enhanced SDT effects (Figure 7A). The *in vivo* study using a xenograft mouse model showed that the prodrug nanoparticles significantly suppressed tumor growth under ultrasound irradiation without obvious adverse effects (Figure 7B).

**FIGURE 8:**
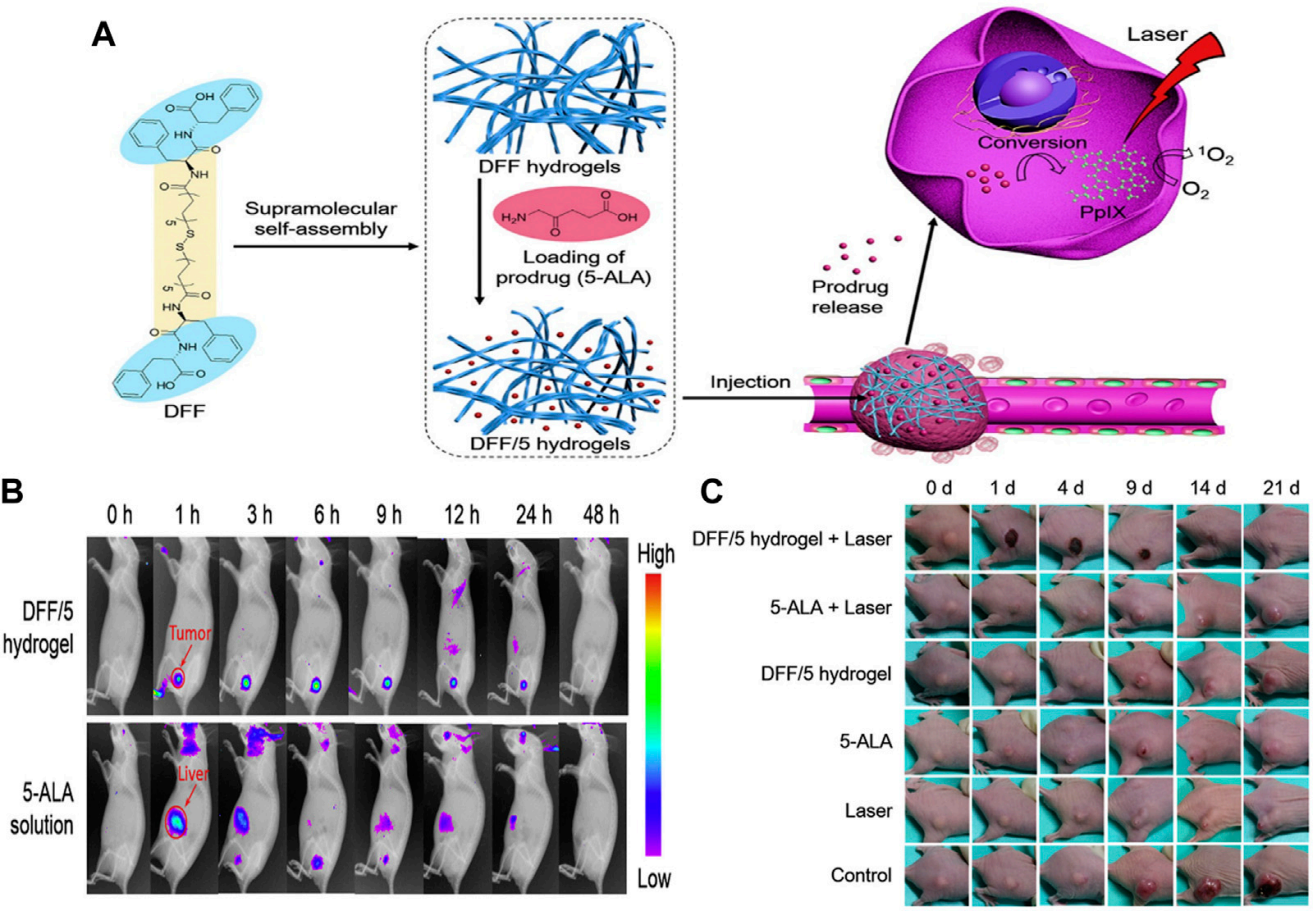
**(A)** Design and mechanism of GSH responsive DFF/ALA hydrogels; **(B)** Fluorescence images of mice at different time points after injection of DFF/ALA hydrogels and free ALA; **(C)** Images of tumor-bearing mice at different time points after treatments. Adapted from ref. (Zou et al., 2020). Copyright 2020 Elsevier B.V.

In addition, hydrogels, as a class of structurally controllable and easily degradable supramolecules, have received increasing attention in topical drug delivery (Li and Mooney, 2016). In 2020, Zou and coworkers reported an injectable and self-assembled bola-dipeptide (DFF) hydrogel for sustained and efficient ALA delivery (Zou et al., 2020). The structural unit of DFF is synthesized by coupling two hydrophilic peptides *L*-Phe-*L*-Phe (FF) *via* a disulfide-containing hydrophobic linker, which next encapsulate ALA molecules into the self-assembled hydrogel through electrostatic interactions (Figure 8A). The ALA-loaded DFF hydrogels exhibited robust mechanical properties and sustained ALA-release upon GSH activation. In particular, this ALA-loaded hydrogel can inhibit/reduce off-target leakage, thereby increasing local PpIX conversion, which in turn enhances tumor imaging and tumor ablation (Figures 8B, C). This study thus demonstrates that GSH-responsive hydrogels might be promising nanoplatforms for local and sustained drug delivery for cancer therapy.

#### 2.1.3 Enzyme-responsive systems

Enzymes are biological catalysts that selectively accelerate reactions and play important roles in physiological, pathological and metabolic processes. However, due to the heterogeneity between normal and pathological tissues, different distributions/expressions of certain enzymes are often observed in tissues/cells, making enzymes potential modules for designing stimuli-responsive drugs (Shahriari et al., 2019; Slor et al., 2021).

**FIGURE 9:**
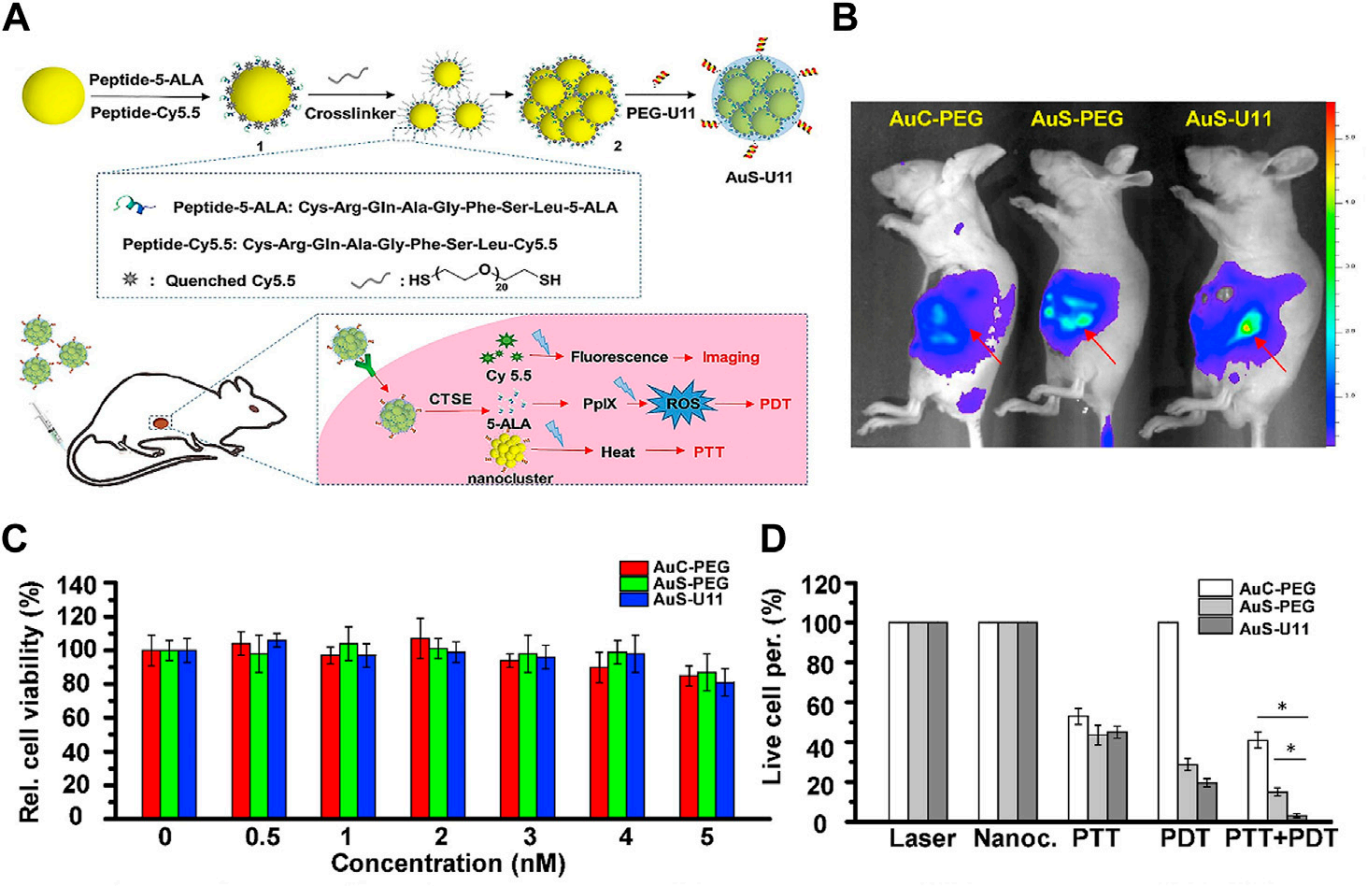
**(A)**. Design and mechanism of CTSE-responsive AuS-U11 prodrug system; **(B)**. *In vivo* images of mice treated with AuS-U11, AuS-PEG and AuC-PEG; **(C)**. Dark toxicities of AuS-U11, AuS-PEG and AuC-PEG against pancreatic ductal adenocarcinoma cells; **(D)**. Cell viabilities of AuS-U11, AuS-PEG and AuC-PEG upon PTT, PDT and combined PTT/PDT treatment under NIR irradiation. Adapted from ref. (Li H et al., 2017). Copyright 2017 Elsevier Ltd.

Cathepsin E (CTSE) is a tumor-associated intracellular enzyme, which is specifically overexpressed in a variety of cancers, including cervical, gastric, intestinal and pancreatic cancer (Dheer et al., 2019). In 2017, Li and coworkers reported a CTSE-triggered nanocluster prodrug (AuS-U11) for endomicroscopy-guided photodynamic and photothermal therapy of pancreatic ductal adenocarcinoma (Li Y et al., 2017). This nanocluster-based prodrug consists of gold nanoclusters as carriers, U11 peptide as a targeting ligand, a CTSE-sensitive PDT prodrug Cys-Arg-Gln-Ala-Gly-Phe-Ser-Leu-ALA (CRQAGFSL-ALA) and a CTSE-sensitive imaging agent Cys-Arg-Gln-Ala-Gly-Phe-Ser-Leu-Cy5.5 (CRQAGFSL-Cy5.5) (Figure 9A). The gold nanoclusters act not only as transporters but also as photothermal agents because the surface plasmon resonance peaks of gold nanoclusters shift to the near-infrared (NIR) region. Especially, this AuS-U11 prodrug demonstrated active targeting ability and enhanced cellular uptake due to the presence of U11 peptide. Stronger signal was observed in the same area under administration of AuS-U11 than that of AuS-PEG (without U11 peptide) and AuC-PEG (without both U11 peptide and ALA) (Figure 9B). Although the nanoclusters (AuS-U11, AuS-PEG and AuC-PEG) alone showed little cytotoxicity (Figure 9C), significant cell-killing effects were observed in AuS-U11 and AuS-PEG-treated group under PDT and PTT treatments. In particular, the AuS-U11-treated group demonstrated the highest cell-killing effects upon both PDT and PTT treatments, suggesting a powerful synergistic antitumor effect (Figure 9D).

**FIGURE 10:**
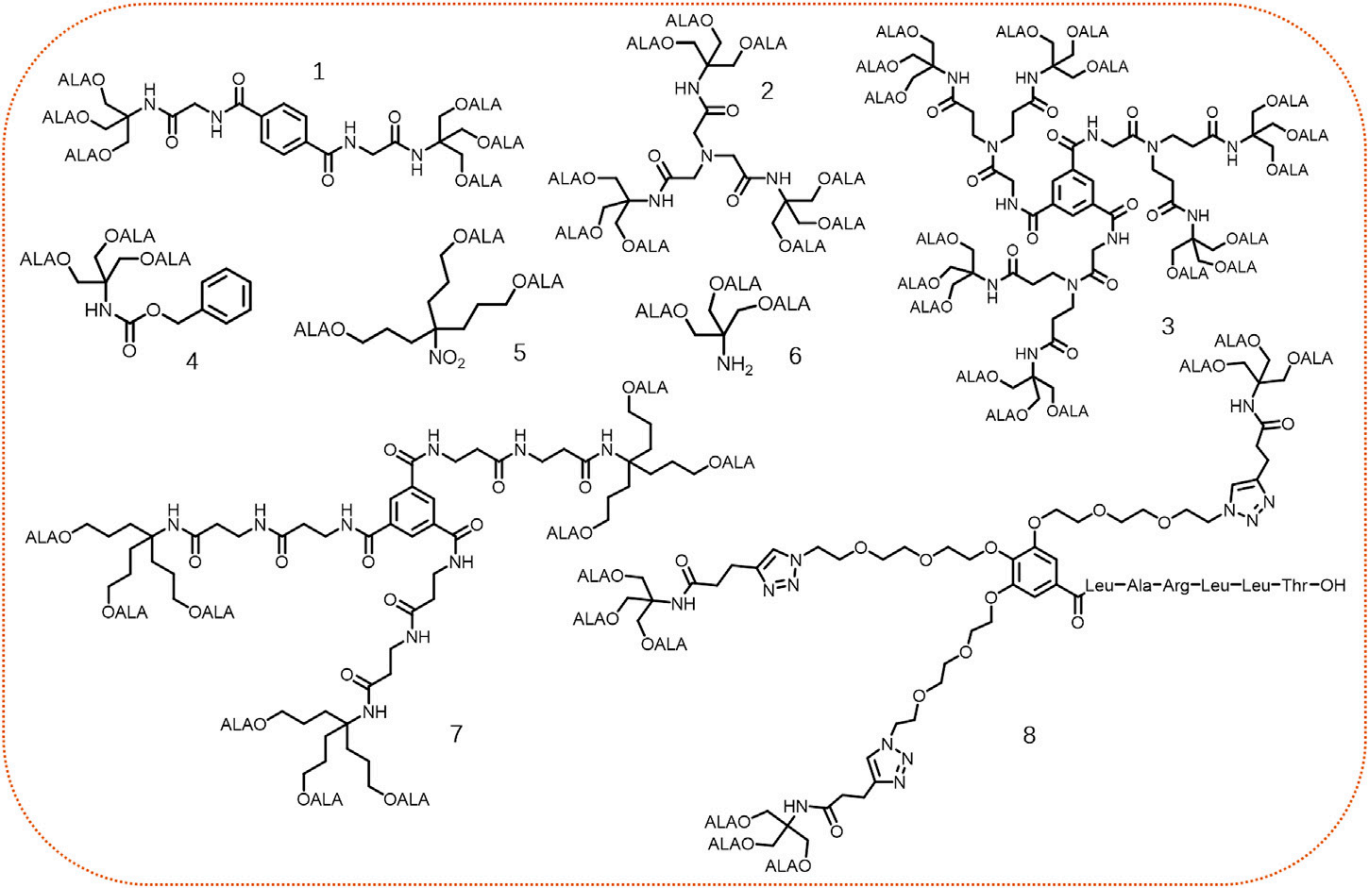
Representative structures of ALA-based dendrimers.

Besides, esterase, another common enzyme overexpressed in certain tumors, plays important roles in tumor growth, progression, invasion, and migration. (Wells and Grandis, 2003). For instance, the activity of esterase in colorectal tumor is around 2–4 folds than in normal tissues (Niu et al., 2012). Dendrimers, as a type of polymeric nanomaterials, possess hyper-branched 3D-structure, high drug loading, and easy control of size and lipophilicity, making them promising nanocarriers in drug delivery (Sherje et al., 2018). Typically, dendrimers constructed by ester linkages are hydrolyzed by intracellular hydrolases (e.g., lysosomal enzymes) to release free monomers. For example, the ALA-containing dendrimers with different core frameworks were synthesized and evaluated by Edwards’s group (Battah et al., 2001). Although these synthesized dendrimers could penetrate cell membrane and release free ALA intracellularly, there was no obvious correlation between the number of conjugated ALA residues in a dendrimer and the subsequent PpIX accumulation (Figure 10, compounds **1–3**). Later, the same group synthesized another three ALA-based dendrimers (Figure 10, compounds **4–6**), which all contained three ALA residues (Battah et al., 2006). The results showed that these three dendrimers produced PpIX more efficiently in cells compared with free ALA. Particularly, compound **5** bearing with a longer alkyl linker was the most effective dendrimer to generate PpIX, most likely due to its greater lipophilicity and less steric hindrance, suggesting lipophilicity and steric hindrance of ALA-based dendrimers might be the key factors affecting ALA release in cells.

Recently, dendrimers **7** (Rodriguez et al., 2015) and **8** (Tewari et al., 2021) both containing 9 ALA residues *via* different linkers were reported for PDT (Figure 10, compounds **7–8**). In contrast with the equimolar amount of free ALA-treated group, more PpIX production was observed in the group treated with ALA dendrimers (**7** or **8**). Notably, dendrimer **8** modified with a targeting peptide sequence demonstrated outstanding cell and tissue selectivity. However, most of the current ALA-based dendrimeric prodrugs mainly focus on the preparation and *in vitro* cell studies, further *in vivo* studies are highly needed to verify the effectiveness of dendrimer-based prodrug strategy.

#### 2.1.4 Light-responsive systems

**FIGURE 11:**
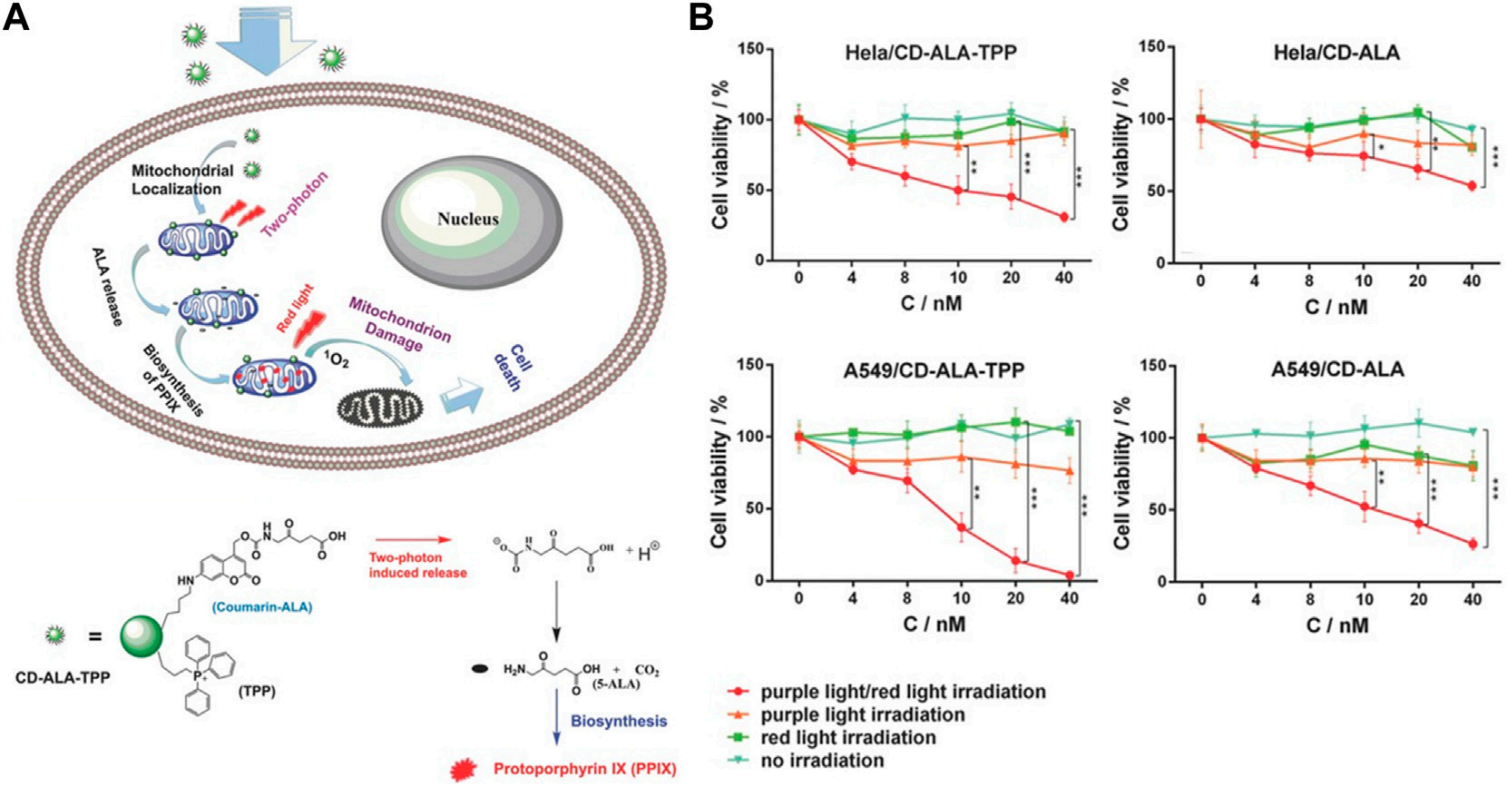
**(A)**. Design and mechanism of light-responsive CD-ALA-TPP nanoprodrug; **(B)**. Cell viabilities of the TPP targeted and non-targeted nanosystems against HeLa and A549 cells. Adapted from ref. (Wu et al., 2016). Copyright 2015 WILEY-VCH Verlag GmbH & Co. KGaA, Weinheim.

Light as an external stimulus with non-invasiveness, ease of use and high spatiotemporal control, has been used in light-responsive drug delivery systems for on-demand drug release (Linsley and Wu, 2017). In 2016, Wu and coworkers reported a mitochondria-targeted nanosystem capable of releasing ALA under two-photon irradiation to induce cell death (Wu et al., 2016). This nanosystem was constructed by covalently attaching a photo-triggerable coumarin-ALA conjugate and a mitochondria-targeting triphenylphosphonium (TPP) ligand onto carbon dots (Figure 11A). After internalization by tumor cells, the prodrugs preferentially accumulate in mitochondria due to the presence of TPP, and release ALA molecules under two-photo irradiation, which are next converted into PpIX through enzyme-catalyzed biotransformation. The *in vitro* study indicated that this nanosystem significantly suppressed tumor cell growth under light irradiation. In particular, the TPP-modified nanosystem demonstrated stronger cell-killing effects than the unmodified agent (Figure 11B), indicating mitochondria-targeting provides enhanced PDT effects.

#### 2.1.5 Multi-stimuli-responsive systems

**FIGURE 12:**
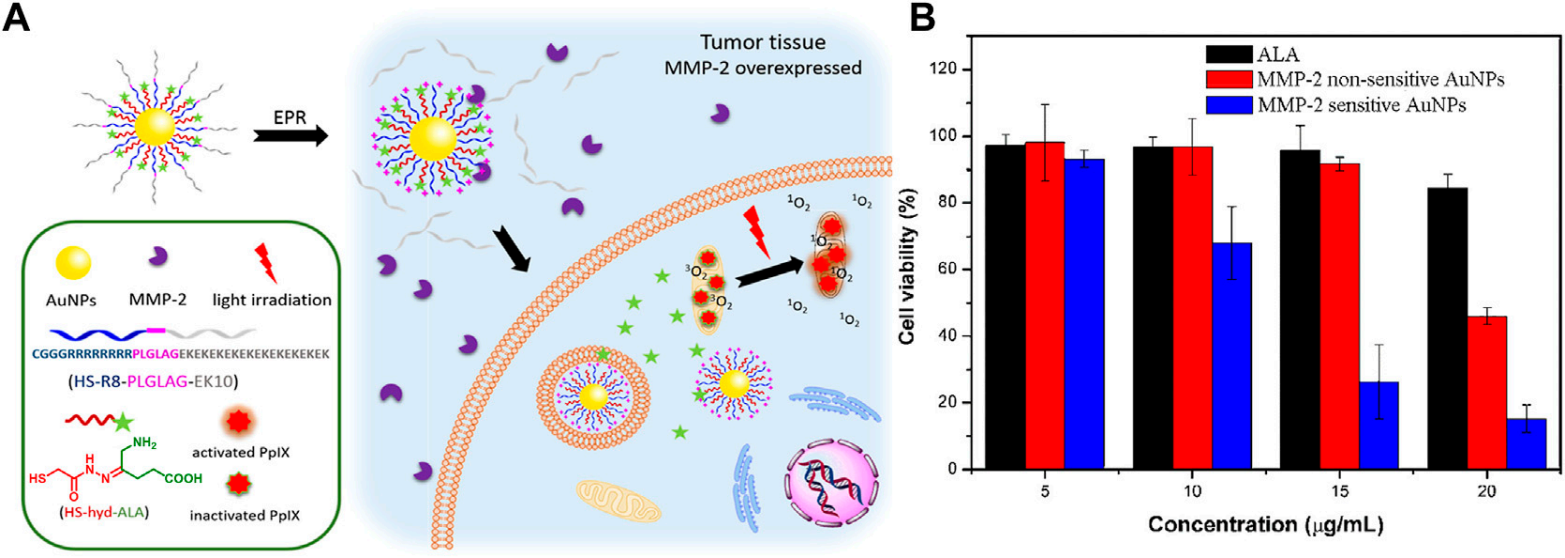
**(A)**. Design and mechanism of pH and MMP-2 dual-responsive AuNPs; **(B)**. Cell viabilities treated with free ALA, MMP-2-sensitive and MMP-2-nonsensitive AuNPs under light irradiation. Adapted from ref. (Wu et al., 2017b). Copyright 2017 American Chemical Society.

Simultaneous responses to more than one stimulus can greatly enhance the selectivity in drug delivery. In 2017, Ji’s group reported a pH-responsive AuNP for targeted delivery of ALA to kill A549 cancer cells (Wu et al., 2017b), and they later reported a pH and matrix metalloproteinase-2 (MMP-2) dual-responsive ALA prodrug nanoparticle for photodynamic killing of SCC-7 cancer cells (Wu et al., 2017a). This dual-responsive nanosystem was fabricated by connecting hydrazone-linked ALA and MMP-2-activatable cell-penetrating peptide (CPP) to AuNPs *via* Au-thiol interaction (Figure 12A). After being taken up by tumor cells, the shield CPPs attached on the nanosystem were removed by overexpressed MMP-2 in tumor cells, and the ALA molecules were liberated through cleavage the hydrazine bonds at an acidic environment in tumor cells. MMP-2-activatable CPP on the nanosystem can enhance cellular uptake, thereby increasing ALA accumulation and generating more PpIX in cells, which in turn results in enhanced PDT effects upon light irradiation (Figure 12B).

**FIGURE 13:**
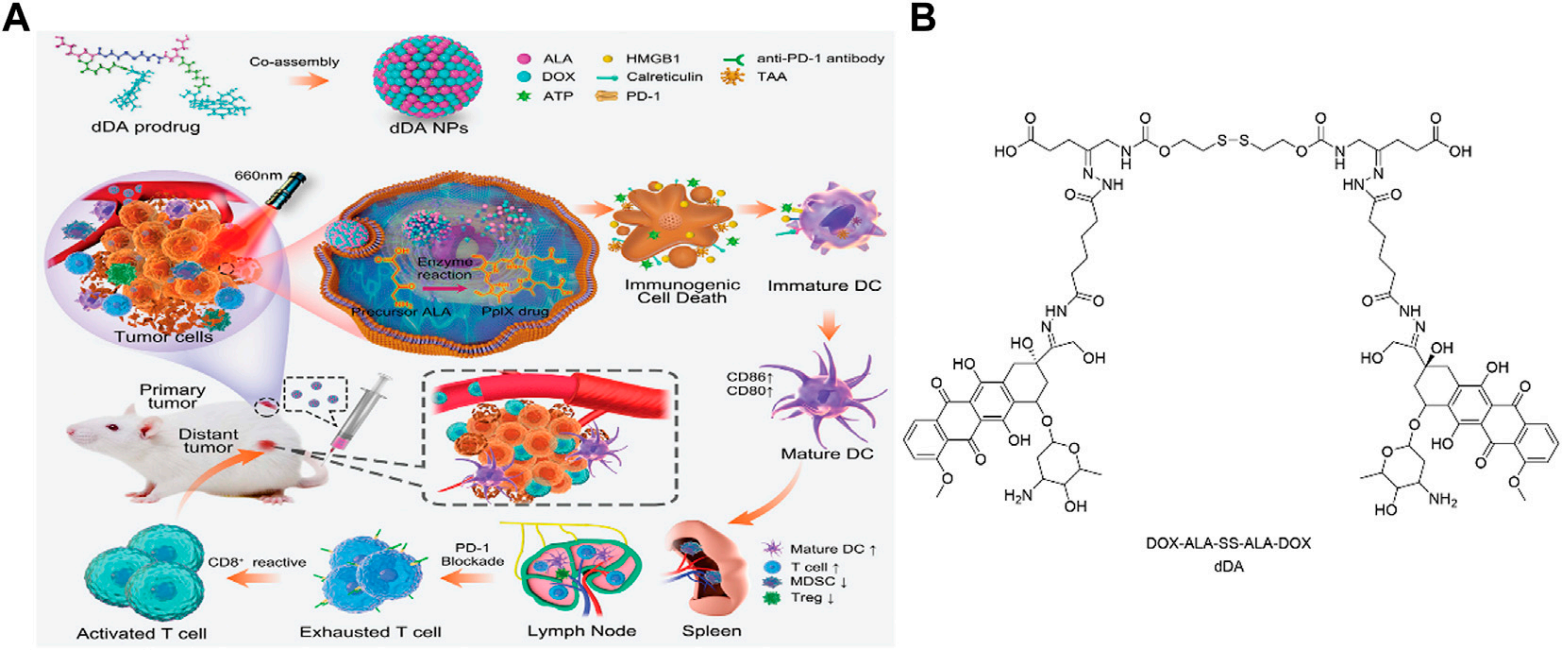
**(A)**. Design and mechanism of pH/GSH dual responsive dDA nanoparticles; **(B)**. Molecular structure of dDA. Adapted from ref. (Bai et al., 2020). Copyright 2020 WILEY-VCH Verlag GmbH & Co. KGaA, Weinheim.

In 2020, Bai and coworkers reported pH/GSH dual-responsive prodrug nanoparticles for chemo-immunotherapy (Bai et al., 2020). In this work, the immunogenicity inducing drug DOX and photodynamic prodrug ALA were fabricated into one platform (DOX-ALA-SS-ALA-DOX, denoted as dDA) using pH and GSH responsive linker, respectively (Figures 13A, B), which were subsequently self-assembled into dDA nanoparticles *via* hydrophobic interaction. Under acidic (hydrazine bond hydrolysis) and high GSH concentration (disulfide bond cleavage) conditions in tumor cells, free Dox and ALA molecules are released to remodel the tumor microenvironment. This nanoprodrug system not only increases the production of PpIX in tumor cells, but also induces immunogenic cell death (ICD) and reduces the number of immunosuppressive immune cells, thereby improving the therapeutic efficacy.

### 2.2 Non-responsive ALA nanoprodrugs

Non-responsive nanoprodrugs refer to prodrugs that are insensitive to external and endogenous stimuli. Generally, molecules encapsulated in nanocarriers are physically absorbed through intermolecular interactions such as hydrophobic interactions, H-bonds, van der Waals forces, electrostatic interactions and dipole-dipole interactions (Li X et al., 2020). After selective uptake by tumor cells through the EPR effect, the nanocarrier-encapsulated therapeutic drugs are released to the target site through natural leakage (Patra et al., 2018). Compared with responsive nanoprodrugs, non-responsive nanoprodrugs are relatively primitive, by which the transported cargos are physically encapsulated into nanocarriers including inorganic nanoparticles, organic nanoparticles, lipid-based nanocarriers and polymer-based nanocarriers. As a hydrophilic prodrug at physiological pH, ALA is encapsulated by nanocarriers mainly through electrostatic/ionic interaction.

Gold nanoparticles (AuNPs), a conventional type of inorganic nanoparticles used in drug delivery and biomedical imaging, possess outstanding drug loading and unique optical properties (Sperling et al., 2008; Cheng et al., 2013). A phase I clinical trial has successfully completed using AuNPs to deliver tumor necrosis factor (TNF), and it showed little systematic toxicity in animal model (Libutti et al., 2010). In 2013, Mohammadi and coworkers reported ALA coated AuNP for enhanced PDT (Mohammadi et al., 2013). In this study, ALA molecules were physically absorbed by AuNPs through electrostatic interactions between the positive-charged AuNPs and negative-charged ALA molecules. Compared with free ALA, AuNP-encapsulated ALA produced more PpIX in Mel-Rm cells, thus inducing a two-fold higher cell death. Similarly, Zhang and coworkers reported ALA-AuNPs-mediated PDT for killing human chronic myeloid leukemia K562 cells (Zhang et al., 2015). This study showed that AuNPs were able to increase the singlet oxygen generation (SOG) *via* a local field enhancement (LFE) effect induced by the localized surface plasmon resonance (LSPR) of AuNPs. However, LFE was dependent on the irradiation wavelength and the size of AuNPs, and the authors revealed that the cell-killing effect was attributed to the delivered ALA rather than the LSPR effect. In 2015, Oliveira *et al.* synthesized PEG-functionalized ALA-AuNPs for atherosclerosis (de Oliveira et al., 2015). PEGylation leads to an enhanced stability of the nanoparticles in aqueous solution and strong resistance to binding against various biomolecules. The PpIX fluorescence extracted from the rabbits’ blood and feces were significantly increased after ALA-AuNPs administration, indicating ALA was efficiently internalized by gold nanoparticles and rapidly transformed into PpIX. This study indicates that ALA-AuNPs might aid in the early diagnosis and treatment of atherosclerosis. Besides, Jiang and coworkers reported gold nanoshells-coated ALA liposome for photothermal-photodynamic antitumor therapy (Jiang et al., 2020). Apart from the photodynamic destruction induced by the delivered ALA, the photothermal effects from gold nanoshells also promoted antitumor effects.

**FIGURE 14:**
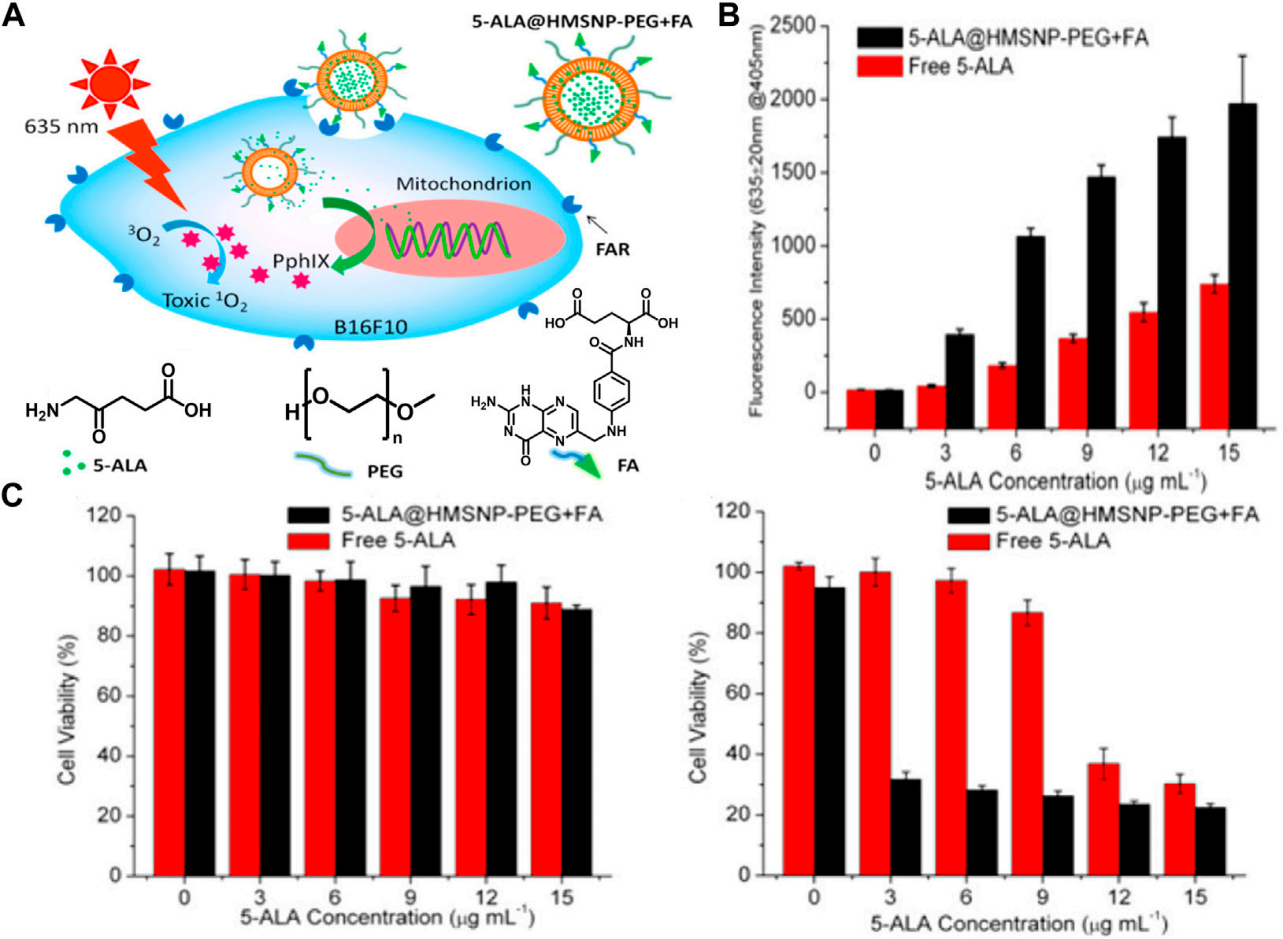
**(A)** Design and mechanism of ALA@HMSNP-PEG + FA; **(B)** PpIX generation in B16F10 cancer cells after treatment with ALA@HMSNP-PEG + FA and free ALA; **(C)** Cell viabilities after treated with ALA@HMSNP-PEG + FA and free ALA without and with light irradiation. Adapted from ref. (Ma et al., 2015). Copyright 2015 American Chemical Society.

Hollow mesoporous silica nanoparticles (HMSNPs) are commonly used organic nanoparticles. The high surface area, good biocompability and high pore volume of HMSNP make them easy to chemically modify and load drugs (Li H et al., 2017). In 2015, Ma and coworkers designed and fabricated a versatile ALA-coated HMSNP for photodynamic treatment of skin cancer (Ma et al., 2015). In this study, folic acid was functionalized onto the nanoparticles, which could target folic acid receptor (FAR) that is overexpressed on the surface of most skin cancer cells and thus facilitate cellular uptake of HMSNP (Figure 14A). In addition, the nanoparticle was further functionalized with PEG to increase biocompatibility and prolong blood circulation. Such fabricated nanoparticles efficiently aid ALA to cross the lipophilic barrier of skin and selectively enter skin cancer cells. By contrast, more PpIX were produced in B16F10 skin cancer cells (Figure 14B) and induced higher PDT effects (Figure 14C) under light irradiation in ALA@HMSNP-PEG + FA-treated group than that of free ALA, suggesting HMSNP-based ALA delivery might be a promising approach in practical skin cancer treatment.

In 2020, Wang et al. constructed a biocompatible periodic mesoporous organosilica coated Prussian blue nanoparticles (PB@PMO) for loading ALA to treat glioma (Wang X et al., 2020). The encapsulated ALA could be delivered into malignant Glima cells and induce more PpIX production compared to free ALA. Besides, the PB core is able to catalyze H_2_O_2_ to generate oxygen in hypoxic tumor microenvironment, thereby attenuating tumor hypoxia and enhancing ALA-mediated PDT. The *in vitro* and *in vivo* results showed that PB@PMO-ALA-mediated PDT effectively suppressed tumor growth, thus providing a promising strategy for local glioma ablation.

Fullerenes (C60), as nanoscale carbon material with distinctive photochemical and electrochemical properties, have been used as drug delivery vehicles in nanomedicine for years (Markovic and Trajkovic, 2008; Torres et al., 2011). In 2014, Li and coworkers first evaluated the *in vitro* and *in vivo* PDT effects of fullerene-loaded ALA (Li et al., 2014). In this study, the prepared C60-ALA nanoparticles were stable in aqueous solution for over weeks, and cells treated with C60-ALA generated more PpIX, thereby inducing enhanced antitumor effect than free ALA in murine tumor model. In addition, Serda and coworkers reported water-soluble C60 nanomaterial modified with D-glucuronic acid for delivery of ALA (C60-GA-ALA) (Serda et al., 2022). Although this strategy led to an enhanced PpIX production in different tumor cells (e.g., MCF-7, HCT116 and A549 cells) upon treatment with C60-GA-ALA, the *in vitro* study using qRT-PCR analysis showed an increased concentration of ABCG2, which might promote PpIX efflux and reduce PDT efficacy. However, the authors verified that this side effect could be efficiently suppressed *via* co-incubation with ABCG2 inhibitor of Ko143.

Due to the unique physiochemical properties, lipids can form various nanomaterials, such as liposome, ethosome and niosome. The architecture of these lipid-based nanomaterials depends on the type of the components and/or the preparation method (Plaza-Oliver et al., 2021; Sguizzato et al., 2021). In 2008, Fang and coworkers compared ALA loaded liposome and ethosome for PDT (Fang et al., 2008). The results showed that the particle size of ethosomes was smaller than that of liposomes, and stronger PpIX fluorescence in the skin was observed for the ethosomes-treated group, suggesting the penetration ability of ethosomes was greater than that of liposomes. Afterwards, the same group used ALA-loaded ethosome to study the recovery of skin in a hyperproliferative murine model (Fang et al., 2009). The results showed that ethosomes penetrated to a depth of approximately 30–80 μm and produced about 3.64 times more PpIX than ALA alone. In addition, Bragagni and coworkers reported ALA loaded niosome for topical PDT treatment of skin malignancies (Bragagni et al., 2015). The penetration depth and drug retention were increased about 80% and 100% for the niosomal formulation compared to free ALA solution. Collectively, lipid-based nanoformulations possess good biocompatibility, high skin permeability and low cytotoxicity, providing potential benefits for ALA-based PDT treatment of skin diseases.

Besides, polymer-based nanomaterials are also frequently used for the delivery of ALA due to their high biological safety, good biodegradability and non-immunogenicity (Han et al., 2018). For instance, Chitosan-encapsulated ALA were used for PDT killing of colon cancer cells and melanoma cancer cells (Chung et al., 2013; Ferreira et al., 2013); poly(lactic-co-glycolic acid) (PLGA) encapsulated ALA were used for PDT killing of skin squamous cell carcinoma cells and 4T1 cells (Shi et al., 2013; de Andrade et al., 2017); and polylactic acid (PLA) -encapaulated ALA were used for PDT killing of HeLa cells (Di Martino et al., 2018). These polymer-based nanomaterials can significantly increase the accumulation of ALA molecules and their subsequent conversion to PpIX, thereby enhancing the PDT efficiency.

## 3 Conclusions and future perspectives

ALA, as a prodrug of PpIX, has been developed nearly 40 years since it was first used to treat skin-associated diseases such as actinic keratoses, basal cell carcinoma, Bowen’s disease, and basosquamous cell carcinoma (Kennedy et al., 1990; Kennedy et al., 1996). Although it is becoming increasingly mature and has been approved by FDA for the treatment of a variety of malignant diseases and for adjuvant surgical resection of high-grade gliomas, the weak lipophilicity, low stability and poor bioavailability of natural ALA reduce its clinical efficiency. Chemical modification of ALA to prepare the esterified derivatives (e.g., MAL, HAL) can improve its stability, tissue penetration and pharmacokinetic properties (Stepp and Waidelich, 2007; Lee and Baron ED, 2011). Besides, the recent emerged nanotechnology has opened another avenue to amplify the effects of ALA-based prodrug owing to the high drug loading, well-controlled drug release, excellent tissue targeting, and the ability to combine multiple treatments into one platform.

In this context, we summarized the important recent advances in the use of various ALA-based prodrug nanomedicines for PDT-related biomedical applications, predominately for cancer therapy (Table 2). Compared with non-responsive ALA nanoprodrugs, design of stimuli-responsive ALA nanoprodrugs may provide a more attractive approach for targeting delivery of ALA. A wide variety of endogenous and exogenous stimuli, as well as a large number of responsive materials capable of fabricating various architectures, provides great flexibility in the design of stimuli-responsive nanosystems. However, most endeavors on stimuli-responsive ALA nanosystems are focused on the *in vitro* studies, only a few have been tested in *vivo* preclinical study, and very few have entered the clinical stage. The difficulty in scaling-up preparation and the complexity in designing the architecture are likely to hamper the translation from bench to bedside. The potential toxicity of the fabricated nanosystem depends on the composition, physiochemical properties and dose. Moreover, lots of stimuli-responsive nanosystems have limited opportunities for translation due to refractory degradation and insufficient biocompatibility. In view of the tremendous work on ALA-based nanoprodrugs, more efforts should be paid on *in vivo* preclinical and clinical studies in future. Particularly, with rapid advances in material science, innovative structure design and multimodal smart responses, future development of easier, simpler, biocompatible and degradable nanosystems, will provide more opportunities to enter the clinical stage.

**TABLE 2:** A summary of ALA-based prodrugs for improved PDT using nanotechnology.

Stimulus	Nanocarrier	Applications	Ref(s)
pH	AuNPs	*in vitro* against A549 cells	Wu et al. (2017a)
	UCNPs	*in vitro and in vivo* against HeLa cells	Punjabi et al. (2014)
	Core/shell-structured nanoparticles	*in vitro* against T24 cells	Tan et al. (2015)
	Gold nanoclusters	*in vitro* against A549 cells	Srinivasulu et al. (2021)
	Quantum dots	*in vitro* against HT29 and SW480 cells	Hashemkhani et al. (2022)
	Micelles	*in vitro* against HepG2 cells	Tong et al. (2016)
GSH	Nanogels	*in vitro and in vivo* against 4T1 cells	Wang Y et al. (2020)
	P-DOA NPs	*in vitro and in vivo* against B16F10 cells	Sun et al. (2022)
	DFF hydrogels	*in vivo* against MCF-7 cells	Zou et al. (2020)
Enzyme	Gold nanoclusters	*in vitro and in vivo* against PANC-1 cells	Li Y et al. (2017)
	Dendrimers	*in vitro* against PAM 212 cells	Battah et al. (2001)
		*in vitro* against PAM 212 cells	Battah et al. (2006)
		*in vitro* against LM3 cells	Rodriguez et al. (2015)
		*in vitro* against MDA-MB-231 cells	Tewari et al. (2021)
Light	Carbon dots	*in vitro* against HeLa and A549 cells	Wu et al. (2016)
pH/Enzyme	AuNPs	*in vitro and in vivo* against SCC-7 cells	Wu et al. (2017a)
pH/GSH	dDA nanoparticles	*in vitro and in vivo* against 4T1 cells	Bai et al. (2020)
Non-stimuli	AuNPs	*in vitro* against Mel-Rm cells	Mohammadi et al. (2013)
		*in vitro* against K562 cells	Zhang et al. (2015)
		atherosclerosis	de Oliveira et al. (2015)
	HMSNPs	*in vitro* against B16F10 cells	Ma et al. (2015)
	PB@PMO nanoparticles	*in vitro and in vivo* against U87MG cells	Wang Y et al. (2020)
	Liposomes	*in vitro and in vivo* against SKOV3 cells	Jiang et al. (2020)
	Ethosomes	*in vivo* for enhancing ALA accumulation/penetration	Fang et al. (2008)
		*in vivo* for enhancing ALA accumulation/penetration	Fang et al. (2009)
	Niosomes	*in vivo* against skin malignancies	Bragagni et al. (2015)
	Fullerenes	*in vitro and in vivo* against B16F10 cells	Li et al. (2014)
		*in vitro* against MCF-7 cells	Serda et al. (2022)
	Polymer-based nanomaterials	*in vitro* against CT26 cells	Chung et al. (2013)
		*in vitro* against B16F10 cells	Ferreira et al. (2013)
		*in vitro* against A431 cells	Shi et al. (2013)
		*in vitro* against 4T1 cells	de Andrade et al. (2017)
		*in vitro* against HeLa cells	Di Martino et al. (2018)
